# Development of a Geropathology Grading Platform for nonhuman primates

**DOI:** 10.31491/apt.2020.03.008

**Published:** 2020

**Authors:** Katie J. Olstad, Denise M. Imai, Rebekah I. Keesler, Rachel Reader, John H. Morrison, Jeffery A. Roberts, John P. Capitanio, Elizabeth S. Didier, Marcelo J. Kuroda, Heather Simmons, Shabnam Salimi, Julie A. Mattison, Yuji Ikeno, Warren Ladiges

**Affiliations:** a California National Primate Research Center, University of California, Davis, CA, USA.; b Comparative Pathology Laboratory, University of California, Davis, CA, USA.; c Wisconsin National Primate Research Center, University of Wisconsin-Madison, Madison, WI, USA.; d School of Medicine, University of Maryland, Baltimore, MD, USA.; e Translational Gerontology Branch, National Institute on Aging, NIH, Dickerson, MD, USA.; f Barshop Institute for Longevity and Aging Studies and Department of Pathology, The University of Texas Health Science Center at San Antonio, San Antonio, TX, USA.; g Department of Comparative Medicine, School of Medicine, University of Washington, Seattle, WA, USA.

**Keywords:** Aging, geropathology, nonhuman primates, age-related diseases, geroscience, rhesus macaques, marmosets, mice

## Abstract

A geropathology grading platform (GGP) for assessing age-related lesions has been established and validated for in inbred strain of mice. Because nonhuman primates (NHPs) share significant similarities in aging and spontaneous chronic diseases with humans, they provide excellent translational value for correlating histopathology with biological and pathological events associated with increasing age. Descriptive age-associated pathology has been described for rhesus macaques and marmosets, but a grading platform similar to the mouse GGP does not exist. The value of these NHP models is enhanced by considerable historical data from clinical, bio-behavioral, and social domains that align with health span in these animals. Successful adaptation of the mouse GGP for NHPs will include 1) expanding the range of organs examined; 2) standardizing necropsy collection, tissue trimming, and descriptive lesion terminology; 3) expanding beyond rhesus macaques and marmosets to include other commonly used NHPs in research; and 4) creating a national resource for age-related pathology to complement the extensive in-life datasets. Adaptation of the GGP to include translational models other than mice will be crucial to advance geropathology designed to enhance aging research.

## Geropathology and the aging human population

Geropathology is defined as the anatomic and molecular assessment of pathology in tissues from aging animals including humans. This report focuses on anatomic geropathology in commonly used animal models for aging research. The need for geropathology and biology of aging research is driven by the rapidly expanding aged human population, with a projected 20 percent increase in people over 65 years of age by 2030 (https://www.census.gov/newsroom/press-releases/2018/cb18-41-population-projections.html). This is in part due to advancements in medical interventions which increase lifespan; however, a longer lifespan is not always associated with a longer health span [1]. According to a 2015 report by the Center for Disease Control, approximately 85% of adults aged 65 years and older are afflicted with at least 1 chronic disease [2], but the relationship between the biology of aging and the initiation of the progression of pathological aging and chronic disease is not well understood. Recently, there has been a paradigm shift in aging research, focusing on enhancing health span rather than lifespan in order to better understand changes that precede the onset of frailty [1]. The determination of health status in human studies is intensive, time-consuming, and complicated by differences in numerous variables including socioeconomic backgrounds and environmental factors. Therefore, development of aging animal models phylogenetically similar to humans is imperative [3].

## Urgent need for geropathology of animal models in aging research

While human indices of aging, such as biomarkers and the frailty index, have successfully been adapted for animal models, anatomic histopathology remains largely underutilized. In 2016, a geropathology working group funded by the National Institute on Aging constructed a descriptive, semi-quantitative, and reproducible histological grading scheme known as the “Geropathology Grading Platform” (GGP) [4]. The GGP was developed to act as a guideline to predict health span by examining clinical and subclinical lesions of aging at the tissue level. The GGP currently uses up to 25 organs and tissues to create semi-quantitative lesion scores utilizing descriptive guidelines generated by the Geropathology Grading Committee [5]. These lesion scores can be summarized into a composite lesion score per tissue or can be dissected by criterion, for example lesion severity, to increase the sensitivity to subtle aging changes. Direct association of composite lesion scores to age, inter-observer repeatability, and alignment with physiologic metrics of aging has been demonstrated in two prototypical mouse strains (C57BL6 and C57BL6 × BALB/c F1) commonly used in aging research [6]. The strength of the platform for laboratory mice lies in the ability to assess response over time in experimental investigations, including genetic manipulation [7] and interventional therapy with a response time as narrow as 2 months [8]. To ensure consistency of this new grading system among different pathologists, proper training is considered crucial. To address this, numerous training tools and venues to complement the GGP have been created, including workshops that offer practice-based learning and a Geropathology Research Network website containing “tissue collection guidelines”, “necropsy protocols”, and a venue for publishing geropathology research notes. A Mouse Geropathology Atlas is in development. Details can be obtained from the Geropathology Research Network website http://www.geropathology.org/.

The laboratory mouse, as an aging animal model, provides numerous advantages such as short lifespan, low price point, large sample size, and ease of environmental and genetic control. While mice develop an array of age-related diseases and lesions, there are variable differences from several common human age-related disease conditions. Therefore, nonhuman primates (NHPs) are considered an excellent translational model as they share many similarities in aging and spontaneous chronic diseases with humans [3, 9]. For example, the interactive nature of cognitive aging, synaptic aging, and endocrine status has been described in NHPs [10], and a relationship between increasing tissue macrophages in heart during aging has been identified [11]. However, there are very few detailed analyses of NHP peripheral organs on how systemic pathologic alterations may be associated with neuronal changes. Archived tissue samples are available for analysis, but there has been no systematic approach to a more comprehensive pathologic survey in studies targeting the brain, even though one would expect cardiac and/or pulmonary health status to contribute to brain health. Studies of HIV-associated aging provide another valuable but underutilized resource. Persons living with HIV have been surviving longer due to the efficacy of anti-retroviral therapy, but they also develop aging-like chronic inflammatory diseases earlier than non-HIV-infected individuals, often referred to as accelerated or accentuated aging [12]. Thus far, studies using SIV-infected rhesus macaques to address accelerated aging and macrophage-associated pathogenesis in lung and gut have demonstrated that increased blood monocyte turnover is associated with increased destruction of shorter-lived, viral infected tissue macrophages [13–16]. Such studies on “inflamm-aging” would benefit from the application of a geropathology grading platform to help better define the mechanisms and impact of macrophages on diseases of aging in specific tissues.

## Geropathology in Rhesus macaques

**Figure 1.**
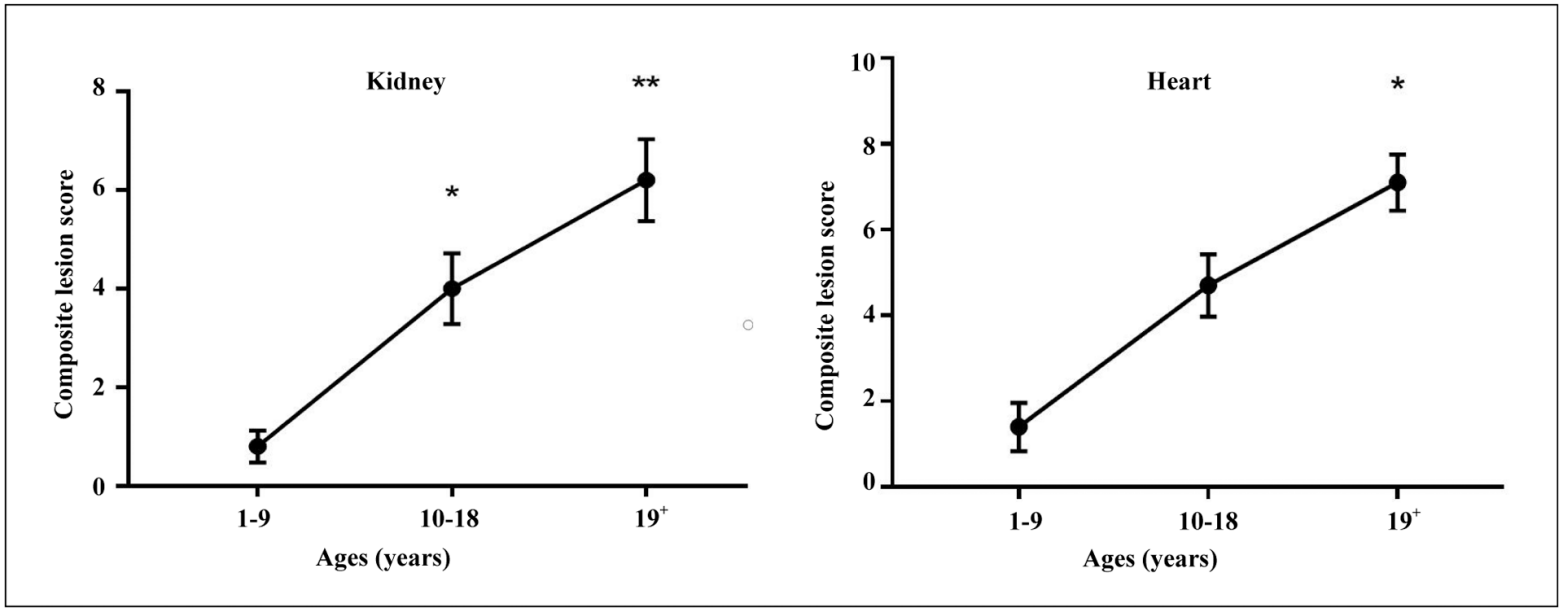
Composite lesion scores (CLS) of kidney and heart from 3 age groups of rhesus macaques increase with age (n = 10/age group). For kidney, CLS was significantly greater in the 10 to 18-year (p = 0.016) and 19+ year (p < 0.0001) age groups than in the 1 to 9-year age group. For heart, CLS was significantly greater in the 19+ year (p = 0.0001) age group than in the 1 to 9-year age group. Kruskal-Wallis Analysis of Variance with Dunn’s multiple comparisons test.

Descriptive age-associated pathology in NHPs has been successfully documented in the literature, but a grading platform similar to the GGP does not yet exist. To gauge the potential utility of a modified NHP GGP in rhesus macaques, two organs, kidney and heart, were examined in 3 different age groups (1–9 years old, 10–18 years old, and 19 years old). Ten animals were assessed per age group separately by two veterinary pathologists. Organ composite scores were created using published aged lesions from each organ as a general guideline [17]. Lesions assessed in the renal composite score included glomerular changes (membranous thickening of Bowman’s capsule, synechiation, and glomerulosclerosis), degree of medullary interstitial accumulations (fibrosis and/or amyloid), degree of infarction, and presence of vascular changes. Lesions assessed in the heart composite score included: degree of interstitial fibrosis, cardiomyocyte degeneration, karyomegaly, and presence of lipofuscin. Composite lesion scores for both organs increased with age (Figure 1) similar to what is seen in mice using the mouse GGP. Although age and composite lesion scores in the heart (Pearson r=0.78, R^2^=0.61, p<0.0001) and the kidney (Pearson r=0.81, R^2^=0.65, p<0.0001) show a positive and strong correlation, extensive work is needed to develop and strengthen a geropathology grading platform adapted for rhesus macaques and nonhuman primates in general.

## A geropathology platform would be valuable for common marmosets

Common marmosets, smaller and shorter lived than rhesus macaques, are a second species of NHPs used in research areas such as infectious diseases, social behavior, cognitive function, and reproduction. There is a growing interest in conducting aging research and intervention of aging and age-related pathology with marmosets. Ross et al [18] recently described lesions in a group of young (average age of 3.3 years) and old (average age of 16.5 years) marmosets. In general, lesions were encountered more frequently in the geriatric cohort, but no descriptions of lesion severity were provided nor was there any indication of how these lesions may have been contributing to the age-related functional decline and overall morbidity of the animals. Thus, it is essential to develop a method to quantitatively assess the severity of the lesions and their impacts on functional changes in various tissues and organ systems. A grading system would enhance these types of observations and provide a semi-quantitative comparison of lesion-based aging.

## Summary and future directions

The successful adaptation of the mouse GGP for NHPs will include (1) expanding the range of organs examined, (2) standardizing necropsy collections, tissue trimming protocols, and descriptive lesion terminology, (3) expanding NHP species beyond rhesus macaques and common marmosets to include other NHPs used in research, and (4) creating a NHP Geropathology Atlas. Adaptation of the GGP to include translational models other than mice will be crucial to advancing geropathology for research communities interested in investigating aging and interventions of age-related diseases.

The value of NHP models is currently enhanced by extensive historical data from a variety of clinical, bio-behavioral and social domains that align with health span in these aged animals. The best example of such a data set is the National Institute on Aging (NIA) Primate Aging Database which includes basic health and husbandry data from multiple institutions on primates across their lifespan. The establishment of a national resource for age related pathology would complement this type of in-life dataset.
